# inVestIgating the pSychologIcal and ecONomic impAct of cataRact surgerY in Vietnam: The VISIONARY observational study protocol

**DOI:** 10.1186/1471-2415-11-25

**Published:** 2011-09-12

**Authors:** Beverley M Essue, Maree L Hackett, Andreas Mueller, Nguyen Thi Hanh Duc, Huynh Tan Phuc, Stephen Jan

**Affiliations:** 1 The George Institute for Global Health; University of Sydney, PO Box M201 Missenden Road, Sydney NSW 2050, Australia; 2 The Fred Hollows Foundation, Level 2 61 Dunning Ave, Rosebery NSW 2018, Australia; 3 The Fred Hollows Foundation (Vietnam), 07 Ton That Dam Street Thanh Khe District, Da Nang City, Vietnam

## Abstract

**Background:**

Visual impairment caused by cataracts not only affects an individual's quality of life but can also have a profound impact on other important psychological factors and on the economic circumstances of individuals and their households. Cataract surgery is an effective intervention to restore vision and is also associated with other positive consequences including improvements in quality of life, economic and psychological outcomes. While there has been an increase in the number and quality of cataract surgeries performed in Vietnam, the programs currently in place are still unable to meet the existing demand and need for surgery. Data on both the cost-effectiveness of cataract surgery and the economic and psychological impact of untreated cataract in this setting is lacking.

**Methods/Design:**

This study, investigating the psychological and economic impact of cataract surgery in Vietnam (VISIONARY), will recruit and interview a sample of adults (18 years or over) who are referred for cataract surgery by one of the following sites and their outreach programs: Hue Eye Hospital; Thai Binh Eye Hospital; Binh Dinh Department of Health Eye Hospital and the Vinh Long Department of Health Social Disease Centre. All participants (those who have cataract surgery and those who do not have surgery) will be followed up at six and 12 months.

**Discussion:**

This study is designed to examine the impact of low vision on household economic circumstances and psychological outcomes as well as to investigate the effectiveness and cost-effectiveness of cataract surgery in Vietnam. It will help to inform international and national non-government organisations working in the country and local policy-makers on priorities for further investment in eye-health services in this setting and their relevance to broader economic development goals.

## Background

Vietnam has a population of 86 million people, of which over two-thirds are between the ages of 15 and 65 years and approximately 5% are over the age of 65 years [1]. While an improving economy over the last few decades has resulted in the creation of new employment opportunities and a dramatic decrease in poverty rates [1], ill-health and poor access to necessary and timely health care continue to pose a threat to the economic wellbeing of individual households and the on-going economic development in this country.

Cataract is the leading cause of blindness in Vietnam, affecting 87.6 new cases per 100,000 each year and causing approximately 65% of all blindness [2]. There is currently a very high prevalence of blindness, affecting 4,034 people per 100,000 [3]. Of even more concern is the staggering numbers who are living with low vision, approximately 18,086 people per 100,000, much of which is caused by untreated cataract [3].

Visual impairment is defined according to the ICD 10^th ^revision 1^st ^and 2^nd ^edition as a visual acuity that is less than 6/18 (equivalent to 3/10) but equal to or better than 3/60, or a corresponding visual field loss of less than 20 degrees in the better eye with best possible correction. Low vision can negatively affect an individual's quality of life [4] and it can also have an impact on other psychological factors such as emotional wellbeing [5]. Furthermore, like other chronic illnesses, vision impairment can have profound economic effects on individuals and their households. This is particularly the case for those from socioeconomically disadvantaged groups and for people with severe disability. As a consequence, chronic illness can be a major cause of poverty due to the impact it has on productive activities such as work, education and caring. In low-income countries such as Vietnam, the economic effects of vision impairment due to cataract can be particularly catastrophic given the absence of social security safety nets.

Cataract surgery has been shown to be effective in restoring vision [4,6] and as a consequence improvements in quality of life, economic and psychological outcomes should to be experienced relatively quickly [5,7,8]. Furthermore, when conducted as part of a regional program, it represents a potentially low cost, high impact intervention that can potentially be rolled out across low income high prevalence settings. There are several international organisations working with local service providers in seeking to improve eye care in Vietnam. The Fred Hollows Foundation, a non-profit international development organisation involved in blindness prevention operates The Vietnam Blindness Prevention Program in ten provinces in both rural and urban areas in Vietnam. This program has contributed to increasing the number and quality of cataract surgeries performed in Vietnam each year. In 2009, the program screened 810,148 people and performed 27,331 sight restoring or improving interventions, which included 11,383 cataract surgeries. ORBIS is another non-profit organisation that is working to eliminate avoidable blindness in the region. In 2008 they screened over 900,000 people and as a result, were able to offer non-surgical ophthalmic medical treatments to over 96,000 people and perform over 26,000 eye surgeries in Vietnam [2].

While there has been an increase in the number and quality of cataract surgeries performed in the last few decades, the programs currently in place are nonetheless still unable to meet the existing demand and need for surgery in Vietnam. A 2009 study of the impact of resources and socioeconomic factors on eye care and blindness prevention, conducted by the Health Strategy and Policy Institute of Vietnam, concluded that a national strategy and action plan for blindness prevention was urgently required and that current networks, training, facilities and budgets were insufficient to meet the current need [9]. Data on both the cost-effectiveness of cataract surgery and the economic and psychological impact of untreated cataract will help to inform priority-setting, health care planning and further investment in eye-health services in this country.

This study will examine the household economic impact and psychological outcomes associated with low vision and will investigate the cost-effectiveness of cataract surgery.

## Methods/Design

### Overview

**Figure 1:**
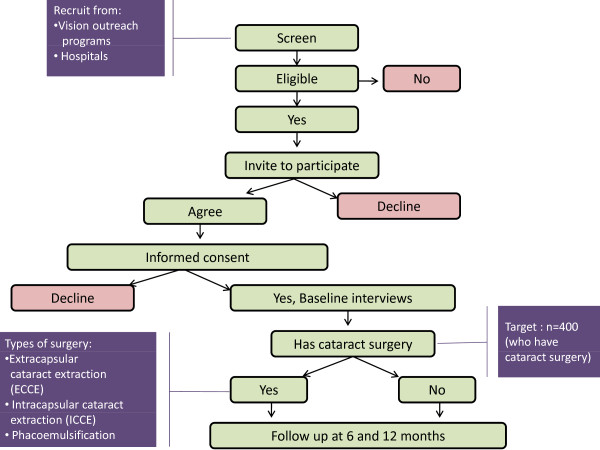
**VISIONARY study design**.

This is a prospective study of individuals who are referred for cataract surgery (Figure 1). The primary aim is to determine the impact that cataract surgery has on quality of life, visual function, household economic circumstances and psychological outcomes. This study will also investigate the cost-effectiveness of cataract surgery.

### Study population

Men and women aged 18 years and older will be eligible to participate in this study if they fulfil the following criteria:

• Receive a referral for cataract surgery, based on a clinical assessment of their best corrected visual acuity for both eyes

○ Visual acuity measured as 3/10 or worse in the better eye

The exclusion criteria are as follows:

• Previous surgery performed to remove a cataract in either eye;

• Another illness of the eye that reduces vision (other than a cataract; does not include blindness caused by injury);

• Unable to participate in two follow up appointments.

### Recruitment

Eye health service providers from each of the participating regions travel to regional areas several times per year to screen people living in the region for vision impairment. Individuals who are screened and referred for cataract surgery will be invited to participate in this study. The eye health service providers will provide eligible individuals with information about the study and ask them if they are interested in joining. Potential participants will be informed that the decision to take part in the study is voluntary, that they are free to withdraw from the study at any time and that whatever their decision, it will not affect their medical treatment or their relationship with the eye health service providers. Individuals who indicate an interest in participating in the study will be referred to the research staff who will obtain written informed consent.

Participants will also be recruited by private health centres that perform cataract surgery in all four regions. Individuals who elect to have the surgery performed privately will present directly to these private health centres. This recruitment strategy will capture the diversity of participants who are living with vision impairment in the regions. To ensure sufficient representation of participants from low socio-demographic backgrounds, the study will only recruit a maximum of one private participant for every participant recruited from the vision outreach programs.

### Study sites

Participants will be recruited from outreach screening programs coordinated by the following sites: Hue Eye Hospital; Thai Binh Eye Hospital; Binh Dinh Department of Health Eye Hospital and the Vinh Long Department of Health and Social Disease Centre.

*Hue Eye Hospital *is located in Thua Thien-Hue, in the Central Region of Vietnam. It has one main city and eight districts and a population of 1.1 million people. The main industries in the region include: tourism, agriculture, forestry and fishing.

Blindness is estimated to affect 1% of the population in this region, with approximately 1,130 new cases per year. 2,200 cataract surgeries are performed in the region per year.

*Thai Binh Eye Hospital *is located in the city of Thai Binh, which is in the Red River Delta, 60 km away from the capital city, Hanoi. Thai Binh has a total population of 1.9 million people. The province is composed of Thai Binh city, and 7 districts. Agriculture is the main industry - 80% of the population are farmers according to national statistics.

Approximately 0.63% of the population are blind or have low vision. In 2009, there were a reported 8,658 people with bilateral blindness and 24,000 people with unilateral blindness due to cataract who needed surgical treatment. About 2,000 cataract surgeries are performed each year in the province.

*Binh Dinh Department of Health Eye *is located in Binh Dinh, in the centre of Vietnam. It has a population of 1.5 million people, most of whom work in the farming or fishing industry.

The leading causes of blindness in the province are cataract, cornea lesions, glaucoma, eye trauma or injury and refractive error. The increasing rate of non-infection eye diseases is one of the challenges of the eye care sector in the province. About 1.1% of the population or 17,400 people are blind. In addition, 7.3% of the population have a visual acuity lower than 1/10. The number of cataract surgeries performed in the region has been steadily increasing in the last three years, with 1,587 surgeries performed in 2008, 1,977 in 2009 and 2,808 in 2010.

*Vinh Long Department of Health Social Disease Centre *is located in Vinh Long, in the Mekong delta; around 120 km away from Ho Chi Minh City. It has a population of 1.1 million people, 57% of whom are farmers.

Approximately 0.63% of the population are blind due to cataracts. The province has eight eye doctors, four of whom are in charge of outreach activities and the remaining four perform treatment activities only.

### Assessments and data collection

The detailed schedule for data collection is shown in Table 1. The interview schedule was developed in English and incorporates questions and tools that have been validated for use in Vietnamese. The full interview schedule has been translated into Vietnamese for administration and piloted with research staff in Vietnam.

**Table 1:** Schedule of assessments in the VISIONARY study

Participant assessment	Baseline	6 months	12 months
Initials	✓		

Full name	✓		

Date of birth	✓		

Contact information	✓		

Next of kin	✓		

***Demographic characteristics***			

Sex	✓		

Age	✓		

Education	✓		

Marital status	✓		

Type of insurance	✓	✓	✓

Household head relationship	✓		

Household composition	✓		

***Employment***			

Main lifetime occupation	✓		

Time spent doing paid work	✓	✓	✓

Time spent doing unpaid work	✓	✓	✓

Impact of eyesight on ability to work	✓	✓	✓

Return to work (paid/unpaid)		✓	✓

Income support	✓	✓	✓

***Household economic situation***			

Annual income	✓	✓	✓

Main source of income	✓	✓	✓

Estimated value of assets (non-livestock and live-stock)	✓	✓	✓

Asset ownership	✓	✓	✓

Financial assistance	✓	✓	✓

***Medical information***			

Visual acuity (in both eyes)	✓	✓	✓

Cataract location	✓	✓	✓

Year of onset of visual impairment	✓		

Co-morbidity	✓	✓	✓

Impact on family carer	✓	✓	✓

Complications from cataract surgery		✓	✓

Visual acuity (in both eyes)	✓	✓	✓

***Health service use***			

Date assessed for cataract surgery	✓		

Date of cataract surgery	✓		

Type of cataract surgery referral	✓		

Type of health service use and number of visits	✓	✓	✓

Out-of-pocket spending	✓	✓	✓

Intention to have cataract surgery	✓	✓	✓

Post-surgery complications		✓	✓

***Quality of life***			

Health related quality of life - SF 12v2 [10]	✓	✓	✓

***Household economic hardship***			

Financial stress	✓	✓	✓

Use of financial coping strategies	✓	✓	✓

Impact of illness on household income	✓	✓	✓

Subjective perception of household economic situation	✓	✓	✓

***Mood (psychological impact)***			

Self-reporting questionnaire (Vietnamese version) [11]	✓	✓	✓

***Screening and interview information***			

Location where consent was obtained	✓		

Interview location	✓	✓	✓

Date of interview	✓	✓	✓

#### Baseline interviews

The baseline interview will be administered in person at the outreach screening program or at the private health centre. Baseline interviews will assess demographic information, medical history, health service use, quality of life, economic circumstances and psychological outcomes. Contact details will be collected from participants at the baseline interview in order to schedule the six and 12 month interviews.

#### 6 and 12 month interviews

A follow-up assessment will be carried out six months and 12 months after the participant's scheduled date of surgery using a similar interview schedule to that administered at baseline. These interviews will be carried out face-to-face in participants' homes or at other locations of their choice. All participants will be followed up, including those who have not had surgery.

### Source of questions included in the assessment tool

#### Quality of life

The primary outcome, health-related quality of life, is measured using The Short Form 12 version 2 Health Survey (SF-12 v2), which includes a subset of questions from The Short Form 36 survey (SF-36). This is a generic tool that uses 12 questions to measure functional health and well-being across the following domains: physical functioning, social functioning, role limitations due to physical problems, role limitations due to emotional problems, mental health, energy and vitality, pain and general health perception [10]. The Vietnamese translation of the SF-36 found that all sub-scales demonstrated good discriminant validity and that the cultural factors specific to the Vietnamese population are not inconsistent with this tool [12]. The questions that correspond to the SF-12v2 will be used in this study.

#### Economic circumstances and health care utilisation

The secondary outcomes, which cover household economic circumstances and health care utilisation, are assessed via the following indicators: employment status; household income; household wealth; household economic hardship and health care utilisation.

To assess *employment status*, questions on time spent in paid and unpaid work for the participant are modified from questions 6-8, 12b and 26-27 from Section 4b of the 2006 Vietnamese Household Living Standards Survey [13]. The average number of hours of paid and unpaid work performed per week over the previous 12 months will be calculated. In addition, at six and 12 months participants are asked to indicate whether, and in what capacity, they have returned to work (paid and unpaid): same job, similar job, different job and the average number of hours worked per week. The date of return to partial or full-time work will be recorded.

The main source of *household income *is modified from questions 12(a-e) in Section 4b of the 2006 Vietnamese Household Living Standards Survey [13]. Participants are also asked about annual income and whether they are receiving any income support payments (sickness, disability, or unemployment payments provided by the Government) or health-insurance benefits.

*Household wealth *is measured using questions that cover key aspects of socioeconomic well-being including details of the dwelling construction and ownership and value of a small number of assets and will be combined into an index of household wealth [14].

*Household economic hardship *will be determined by a series of questions about failure to pay basic living and medical expenses and whether there was help provided by any organisation or individual to meet these payments. An advantage of this measure is that it is sensitive to the possibility that individuals prioritise certain payments (e.g. forgo medical expenses to pay school fees). The basis for these questions comes from the US Census Survey of Income and Program Participation [15] and a modified version has been used in the authors' previous work [16-18]. Economic hardship will be constructed as a dichotomous variable where a reported inability to make any one of the payments posed to respondents will be classed as a case of economic hardship.

*Health service utilisation *and the associated out-of-pocket expenditure for both health and non-health categories is collected using a modified version of Questions 8-12 from Section 3a of the 2006 Vietnamese Household Living Standards Survey [13]. The out-of-pocket burden will be calculated as the total amount spent out-of-pocket as a proportion of annual household income.

#### Psychological impact

The impact of cataracts is greater than the immediate visual impairment and can be associated with psychological co-morbidities such as increased symptoms of anxiety and depression. The third outcome, reductions in depressive, anxiety and somatic symptoms, will be assessed using the self-reporting questionnaire-20 (Vietnamese version) [11]. Each of the 20 questions scores either a zero or one. A score of one indicates that the symptom was present during the past month and a score of 0 indicates that the symptom was absent. The maximum score is 20.

#### Demographic information

Basic demographic information is collected as explanatory variables, including the presence of co-morbidity which is assessed based on self-reported illness episodes [19]. The question asks: How many times in the last 4 weeks have you: (a) stayed in bed or been absent from work for at least one day due to illness; (b) had reduced working capacity (not able to perform usual functions) and (c) used any kind of medical or traditional treatment.

### Statistical considerations

Improvements in the physical functioning domain following cataract surgery have been shown in both an older US and an older Spanish population, 3.2 and 4.0 point improvements respectively [4,20]. The sample size for this study has been calculated on the basis of detecting improvements in this primary outcome measure following cataract surgery. Assuming a lost to follow up rate of 40%, 266 participants would be needed to detect a 4-point difference in the physical functioning domain with 90% power, a 5% two sided significance level, and a standard deviation of 17 [20]. We will oversample in order to allow sufficient numbers to also investigate the secondary outcomes, the household economic and psychological impact of visual impairment, so will recruit a total of 400 individuals who have cataract surgery across the four study sites. The number of participants recruited from each program site will be proportionate to the number of people living in the region and prevalence of visual impairment in the region.

### Statistical analysis

For descriptive purposes, baseline characteristics will be presented for the surgical group (those who have had cataract surgery) and the non-surgical group (those who have not had surgery). Discrete variables will be summarised by frequencies and percentages. Continuous variables will be summarised by use of standard measures of central tendency and dispersion, including median and inter-quartile ranges given potential skewness in the data.

We will calculate mean changes in the SF-12v2 scores for the surgical and non-surgical participants at the six months and one year follow up points. Reductions in scores indicate deterioration and improvements in scores indicate an improvement in quality of life. Generalised linear models will be used to assess multivariate-adjusted mean differences in SF-12v2 scores comparing surgical and non-surgical participants.

To assess the impact of cataract surgery on household economic circumstances and psychological outcomes, models will be constructed based on each of the secondary and tertiary outcomes listed above. For the dichotomous outcomes, 'returned to work', change in household income and economic hardship, the models will be estimated through logistic regression. Household wealth and psychological impact, specified as continuous outcomes, will be modelled using parametric or non-parametric methods depending on the distributional characteristics of each outcome. The modelling will account for the potential random effects, as each individual respondent will yield multiple measures.

The cost-effectiveness analysis will take a societal perspective. The costs of surgery will be assessed through financial statements provided by Vietnam Blindness Prevention Program staff. These will include staff, travel and training costs associated with running the cataract surgery program, including the costs of the operations. Cost-offsets in terms of use of health care services and medications will be assessed based on individual self-report in the follow up interviews. Effectiveness will be assessed in terms of quality of life, using the SF-12v2, with incremental cost-effectiveness ratio estimated based on cost per quality adjusted life years.

### Ethics approval

Each of the participating sites reviewed a copy of the research protocol and provided written approval and agreement to participate in this study.

This study has also been approved by the University of Sydney's Human Research Ethics Committee (Protocol number: 13407).

## Discussion

Evidence on the cost of visual impairment and cost savings associated with preventing visual impairment at a global level can be important advocacy tools for global campaigns for the prevention of blindness (i.e. the VISION 2020 and The Right to Sight campaign). However, global data is only of limited value for advocacy purposes at a country-level and so there is a need for evidence on the economic impact of visual impairment in specific developing countries. Indicators for costs associated with visual impairment, including: the value of lost income and the role of formal health care versus family support are expected to differ widely between developed and developing countries. This study will provide novel and valuable information about the economic impact of visual impairment in a developing country and will further assess the economic benefit associated with the prevention of visual impairment from cataract in this setting.

## Competing interests

The authors declare that they have no competing interests.

## Authors' contributions

BME, MLH, AM and SJ: conceived of and designed the study, including: drafting the protocol and this manuscript and developing all research tools; trained research staff and will perform the statistical analysis. NTHD and HTP participated in the design of the study and its coordination in Vietnam. All authors read and approved the final manuscript.

## Pre-publication history

The pre-publication history for this paper can be accessed here:

http://www.biomedcentral.com/1471-2415/11/25/prepub

## References

[B1] Vietnam country context http://www.wpro.who.int/countries/vtn/2010/VTN.htm

[B2] Country Profile for Vietnam http://www.orbis.org/Default.aspx?cid=8242&lang=1&pre=view

[B3] Keeffe JE Konyama K Taylor HR Vision impairment in the Pacific region British Journal of Ophthalmology 2002 86 605 610 10.1136/bjo.86.6.605 12034678 PMC1771168

[B4] Bilbao A Quintana JM Escobar A García S Andradas E Baré M Elizalde B Group. ftI-C Responsiveness and Clinically Important Differences for the VF-14 Index, SF-36, and Visual Acuity in Patients Undergoing Cataract Surgery Ophthalmology 2009 116 418 424 10.1016/j.ophtha.2008.11.020 19168220

[B5] Walker JG Anstey KJ Hennessy MP Lord SR Von Sanden C The impact of cataract surgery on visual functioning, vision-related disability and psychological distress: a randomized controlled trial Clinical & Experimental Ophthalmology 2006 34 734 742 10.1111/j.1442-9071.2006.01340.x 17073895

[B6] Lamoureux EL Hooper CY Lim L Pallant JF Hunt N Keeffe JE Guymer R Impact of Cataract Surgery on Quality of Life in Patients with Early Age-Related Macular Degeneration Optometry & Vision Science 2007 84 683 688 610.1097/OPX.1090b1013e31812f31755f 10.1097/OPX.0b013e31812f755f 17700333

[B7] Ishii K Kabata T Oshika T The Impact of Cataract Surgery on Cognitive Impairment and Depressive Mental Status in Elderly Patients American Journal of Ophthalmology 2008 146 404 409 10.1016/j.ajo.2008.05.014 18602079

[B8] Kuper H Polack S Mathenge W Eusebio C Wadud Z Rashid M Foster A Does Cataract Surgery Alleviate Poverty? Evidence from a Multi-Centre Intervention Study Conducted in Kenya, the Philippines and Bangladesh PLoS ONE 2010 5 e15431 10.1371/journal.pone.0015431 21085697 PMC2976760

[B9] Impacts of resources and socioeconomic factors on eyecare and blindness prevention and control http://www.hspi.org.vn/vcl/en/home/InfoDetail.jsp?area=1&cat=102&ID=1050

[B10] Quality Metric http://www.qualitymetric.com/

[B11] Giang KB Allebeck P Kullgren G Tuan NV The Vietnamese version of the self reporting questionnaire 20 (SRQ-20) in detecting mental disorders in rural Vietnam: a validation study International Journal of Social Psychiatry 2006 52 175 184 10.1177/0020764006061251 16615249

[B12] Watkins RE Plant AJ Sang D O'Rourke T Gushulak B Development of a Vietnamese Version of the Short Form-36 Health Survey Asia Pacific Journal of Public Health 2000 12 118 123 10.1177/101053950001200211 11841039

[B13] Vietnamese Household Living Standards Survey, 5th round - DDI-VNM-VHLSS-2006-v1.0 http://www.gso.gov.vn/nada2/index.php?page=catalog

[B14] Hargreaves J Morison L Gear J Kim J Makhubele M Porter J Watts C Pronyk P Assessing household wealth in health studies in developing countries: a comparison of participatory wealth ranking and survey techniques from rural South Africa Emerging themes in epidemiology 2007 4 4 10.1186/1742-7622-4-4 17543098 PMC1894790

[B15] Survey of Income and Program Participation (SIPP) http://www.census.gov/sipp/

[B16] Essue B Kelly P Roberts M Leeder S Jan S We can't afford my chronic illness! The out-of-pocket burden associated with managing chronic obstructive pulmonary disease in Western Sydney, Australia Journal of Health Services Research and Policy 2011 in press Accepted: 30 March 2011 10.1258/jhsrp.2011.010159 21954233

[B17] Hackett M Glozier N Martiniuk A Jan S Anderson C Sydney epilepsy incidence study to measure illness consequences: the SESIMIC observational epilepsy study protocol BMC Neurology 2011 11 3 10.1186/1471-2377-11-3 21214957 PMC3025848

[B18] Hackett ML Carter G Crimmins D Clarke T Maddock K Sturm JW imProving Outcomes after STroke clinical pilot trial protocol International Journal of Stroke 2010 5 52 56 20088995 10.1111/j.1747-4949.2009.00388.x

[B19] Giang KB Allebeck P Self-reported illness and use of health services in a rural district of Vietnam: findings from an epidemiological field laboratory Scandinavian Journal of Public Health 2003 31 52 58 10.1080/14034950310015112 14649642

[B20] Mangione CM Phillips RS Lawrence MG Seddon JM Orav EJ Goldman L Improved visual function and attenuation of declines in health-related quality of life after cataract extraction Arch Ophthalmol 1994 112 1419 1425 7980131 10.1001/archopht.1994.01090230033017

